# Gene-Environmental Interactions as Metabolic Drivers of Nonalcoholic Steatohepatitis

**DOI:** 10.3389/fendo.2021.665987

**Published:** 2021-05-10

**Authors:** Somaya Albhaisi, Arun J. Sanyal

**Affiliations:** ^1^ Department of Internal Medicine, Virginia Commonwealth University, Richmond, VA, United States; ^2^ Division of Gastroenterology, Hepatology and Nutrition, Department of Internal Medicine, Virginia Commonwealth University, Richmond, VA, United States

**Keywords:** NASH, metabolic syndrome, genes, insulin resistance, lipotoxicity, oxidative stress, inflammation, gut microbiome

## Abstract

Nonalcoholic fatty liver disease (NAFLD) has emerged as a leading cause of chronic liver disease worldwide in the past few decades as a consequence of the global obesity epidemic and is associated with significant morbidity and mortality. NAFLD is closely associated with components of the metabolic syndrome, type 2 diabetes mellitus and cardiovascular disease, suggesting a plausible metabolic mechanistic basis. Metabolic inflexibility is considered a nidus for NAFLD pathogenesis, causing lipotoxicity, mitochondrial dysfunction and cellular stress leading to inflammation, apoptosis and fibrogenesis, thus mediating disease progression into nonalcoholic steatohepatitis (NASH) and ultimately cirrhosis. In this review, we describe they key metabolic drivers that contribute to development of NAFLD and NASH, and we explain how NASH is a metabolic disease. Understanding the metabolic basis of NASH is crucial for the prevention and treatment of this disease.

## Introduction

Non-alcoholic fatty liver disease (NAFLD) has become one of the most common causes of chronic liver disease worldwide and is rapidly becoming the most common indication for liver transplantation (1, 2). It is a major public health problem of growing prevalence globally paralleling the increase in the prevalence of obesity. NAFLD is often a progressive disease associated with multifaceted deleterious impact and significant complications such as cirrhosis, end-stage liver disease, hepatocellular carcinoma (HCC), and increased overall mortality (3). Nonalcoholic steatohepatitis (NASH) is the inflammatory subtype of NAFLD and is associated with steatosis, inflammation, hepatocyte injury, and that can progress to cirrhosis (4). The understanding of the pathophysiology of NASH has evolved substantially. A large body of evidence strongly supports that NAFLD is the hepatic manifestation of the metabolic syndrome, with insulin resistance being the common driving factor (5). It became clear that NAFLD is a complex multisystem disorder with significant clinical and pathogenic heterogeneity. There is a variety of underlying mechanisms for its development, with the dominant driver being alterations in hepatic and extra-hepatic lipid metabolism (6, 7). The disease susceptibility and progression are likely attributed to dynamic interactions between genetic and environmental factors (8–10). Variations in genetic background have been identified as an underlying etiology for the interindividual variability in the natural history of the disease (11–13). Epigenetic alterations that occur in response to environmental factors also contribute to the development of NAFLD (10). It has been shown that only a proportion of patients develop chronic inflammation (14). Thus, a small subset of patients with NAFLD develop advanced fibrosis or cirrhosis. Overall, only a minority only experience associated liver-related morbidity (15, 16). In addition, not all obese individuals will develop NAFLD and, more importantly, NAFLD can develop in non-obese individuals (an entity known as ‘Lean NAFLD’) (17). Also, several studies indicate strong heritability of hepatic fat content (11). Therefore, there has been an increased focus in the last few years on exploring genetic factors associated with NAFLD.

## Diet and The Extrahepatic Milieu

Strong evidence has shown that the metabolic basis of NAFLD is a part of a metabolic disease cluster due to its association with obesity, insulin resistance, type 2 diabetes mellitus (T2DM), hyperlipidemia and cardiovascular disease (CVD) (18). Individuals with NAFLD or NASH typically have hepatic and adipose tissue insulin resistance, with hyperinsulinemia demonstrated even in presence of normal glucose tolerance (19–21). High‐calorie diets, excessive consumption of sugar and sedentary lifestyle predispose to NAFLD and NASH and have been linked to the development of other components of the metabolic syndrome (22–24). A common feature in the Western diet is increased fat and fructose consumption that is promoting obesity and fatty liver (7). Diet and diet-related adiposity remain a major cause of NAFLD. Diet can impact the development of NAFLD by promoting obesity and excess adipose tissue which can become inflamed (25). It can also alter the intestinal microbiome and alter intestinal permeability thus increasing systemic exposure to microbial products that are normally largely excluded such as endotoxin resulting in activation of the innate immune system and driving a systemic inflammatory state (26, 27). Diet can also contribute to the load of free fatty acids to the liver both directly and by promoting insulin resistance (28). Intake of sugars can contribute by both associated glucotoxicity and by serving as a substrate for de novo lipogenesis. Fructose consumption (such as via sugared sweetened beverages) is associated with extensive metabolic dysfunction, including insulin resistance, altered gut microbiota, dysregulated lipid metabolism and hepatic steatosis (29). Fructose further bypasses several regulated steps in hexose metabolism and can directly serve as a substrate for de novo lipogenesis (30, 31); this has led to efforts to use ketohexokinase inhibitors as a treatment of NASH (NCT03248882) (32, 33).

In contrast, the Mediterranean diet plays a beneficial role in reducing liver fat and improving cardiovascular risk in patients with NAFLD (34). This diet is enriched in n3 and n6 polyunsaturated fatty acids (PUFAs). N3 PUFAs have insulin-sensitizing, anti-inflammatory effects and also impact membrane function across many organ systems. They further reduce hypertriglyceridemia and are expected to improve NAFLD. There are however no long term controlled data to support this as a stand-alone approach to managing the patient with NASH. Also, clinical trials of n3 PUFAs have been disappointing (35). Whether the benefits of such a diet are realized only in the context of the Mediterranean or Ikaria lifestyle or in all settings remains to be established. Further, this diet as all other diets must be considered in the relevant social-cultural context.

**Figure 1:**
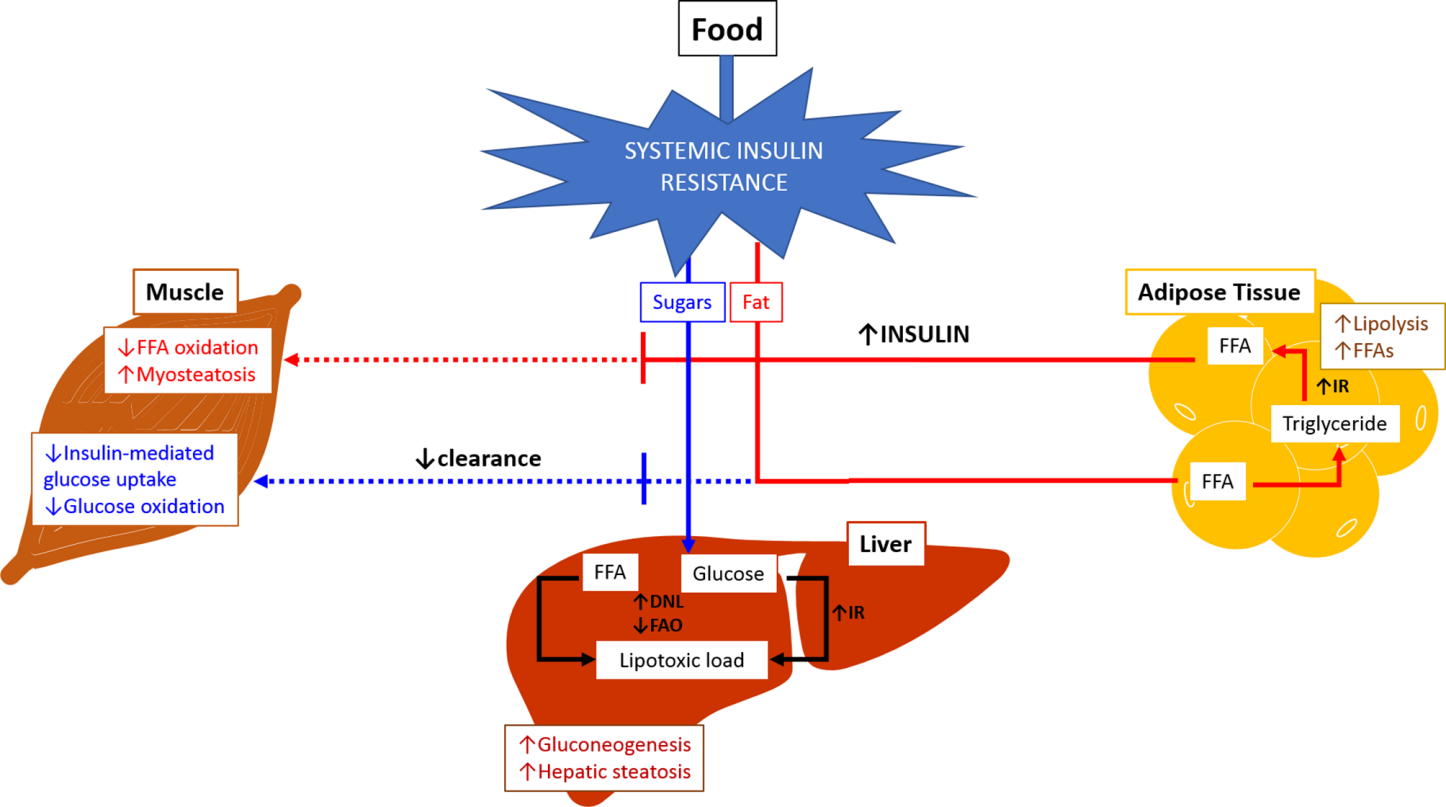
Metabolic Inflexibility. In physiological conditions, fasting state is associated with relatively low insulin levels leading to increased lipolysis and free fatty acid oxidation in adipose tissue and muscles. Glucose oxidation increased in muscles, and gluconeogenesis is activated in the liver. During fed state, food intake increases insulin release which subsequently stimulates lipogenesis and triglyceride accumulation in adipose tissue. There is increase in glucose and free fatty acid oxidation in muscles, and inhibition of gluconeogenesis in the liver. Inability of the body to maintain this balance or to adequately handle substrates at appropriate times is referred to as metabolic inflexibility. The liver loses its ability to flexibly switch back and forth between prandial and fasting states due to exacerbated insulin resistance (hallmark of NAFLD/NASH). Metabolic inflexibility is associated with hyperinsulinemia, systemic lipotoxic cell stress leading to inflammation and fibrogenesis, and eventually NASH. (Adapted from Chakravarthy MV, Siddiqui MS, Forsgren MF and Sanyal AJ (2020), Harnessing Muscle–Liver Crosstalk to Treat Nonalcoholic Steatohepatitis. *Front. Endocrinol*. 11:592373. doi: 10.3389/fendo.2020.592373.). DNL, de novo lipogenesis; FAO, fatty acid oxidation; FFA, free fatty acid; IR, insulin resistance.

Metabolic flexibility refers to the body’s ability to adequately handle substrates and maintain energy homeostasis (**Figure 1**) (36). The inability to handle substrates and calories appropriately is referred to as metabolic inflexibility which tips the energy balance scale towards higher intake and storage, leading to lipotoxic cell stress. Metabolic inflexibility has been recognized as a driving factor for dysregulation of energy homeostasis typically seen in NAFLD/NASH and contributes to dysregulated glucose and lipid metabolism resulting in insulin resistance and dyslipidemia (37). The persistently high intake of sugar and fat on a background of obesity and insulin resistance results in the inability to store free fatty acids (FFAs) in the adipose tissue in addition to increased triglycerides (TG) lipolysis into FFAs. The muscle responds by reducing fat and glucose oxidation.

The resultant high influx of FFAs to the liver combined with increased de novo lipogenesis leads to lipotoxic stress. The process of conversion of FFAs to TG within hepatocytes inhibits peroxisome proliferator‐activated receptor‐α (PPARα) signalling (38) and thus maintaining intrahepatic FFA accumulation. Other key signalling pathways mediated by c-Jun NH2-terminal kinase (JNK) (39), toll-like receptors (TLR4) (40) and novel protein kinase C isoform PKCϵ (41) exacerbate hepatic insulin resistance leading to a vicious cycle of increased FFA, TG accumulation and formation of toxic lipid species that cause cellular stress (42–44). Overall, these processes contribute to high circulating insulin levels and FFA and inappropriate fat deposition. The hyperinsulinemia and lipotoxic stress act as key triggers for the development of NAFLD. Presence of metabolic syndrome, T2DM and other factors can further heighten the impact of insulin resistance and lipotoxicity which consequently impair the repair response of the liver resulting in progression of the disease (44, 45).

## The Microbiome as A Driver of Dysmetabolic State

There is growing body of evidence supporting the role of gut microbiome in the development and progression of NAFLD (**Table 1**) (52, 53). Quantitative and qualitative alterations of gut microbiota composition (also known as ‘Dysbiosis’) have been recognized in patients with NAFLD and NASH. Instances of gut microbiota producing hepatotoxic substances such as ethanol which alter gut permeability and thus inducing endotoxemia secondary to translocation of bacterial products have been reported (54, 55). Patients with NASH tend to have increased levels of circulating endotoxins that can activate innate immune responses and consequently exacerbating the disease (56). Circulating pathogen-associated molecular pattern (PAMPs) and damage-associated molecular patterns (DAMPs) activate TLRs within the liver and induce production of pro-inflammatory cytokines and reactive oxygen species and activation of the inflammasome (57, 58). Several studies demonstrated that NAFLD was associated with increased Firmicutes/Bacteroidetes ratio and that specific metagenomic signatures in the intestinal microbiota are robust predictors of advanced fibrosis associated with NASH (46, 59, 60). Gut microbiota are also implicated in modulating bile acid-related pathways, which sequentially regulate lipid and carbohydrate metabolism as well as energy homeostasis (61). Recently, there has been growing interest in evaluating the influence of human genetic variation and ethnicity in driving gut microbiota diversity (62–64). However, separating the effects of diet on liver health from the effects of diet-induced microbiota changes and its role in driving risk for liver disease, remains challenging. Furthermore, most of the literature has focused on associations and mechanistic studies are now needed to better understand how the microbiome leads to NAFLD and contributes to its progression.

**Table 1:** Examples of human studies on the gut microbiota-derived metabolites in NAFLD and NASH.

Study	Subjects	Type of metabolites	Type of sample	Results
Loomba et al. (46)	Adults with NAFLD (n = 86)	Short Chain Fatty Acids	Blood	↑abundance of enzymes associated with lactate, acetate, and formate in mild/moderate NAFLD. ↑abundance of enzymes for butyrate, D-lactate, propionate, and succinate in advanced fibrosis. ↑serum 3-phenylpropanoate in advanced fibrosis.
Hoyles et al. (47)	Morbidly obese women (n = 105)	Amino Acids	Blood	↑Phenylacetic acid, ↑ Valine, ↑Leucine, ↑ Isoleucine
Mouzaki et al. (48)	Adults with NASH (n = 22), SS (n = 11), healthy controls (n = 17)	Bile Acids	Fecal	↑Primary to secondary BA ratio in NASH
Raman et al. (49)	Adults with NAFLD (n = 30), healthy controls (n = 30)	Ethanol, VOCs	Fecal	↑Butanoic acid, ↑Propanoic acid, ↑Acetic acid, and ↓2-butanone in NAFLD vs. healthy controls
Da Silva et al. (50)	Adults with SS (n = 15), NASH (n = 24), healthy controls (n = 28)	Short Chain Fatty Acids	Fecal and Blood	(Fecal) ↑Isobutyric acid, ↑Propionate (Serum) ↑2-hydroxy-butyrate, ↑L-lactic acid
Del Chierico et al. (51)	Children and adolescents (n = 61) with NAFLD, NASH, or obesity, healthy controls (n = 54)	VOCs	Blood	↑2-butanone and ↑1-pentanol in NAFLD. ↑2-butanone and ↑4-methyl-2-pentanone in NASH

↑, increase; ↓, decrease; NAFLD, nonalcoholic fatty liver disease; NASH, nonalcoholic steatohepatitis; SS, simple steatosis; BA, bile acids; VOC, volatile organic compounds.

## Sarcopenia as a Driver of Nash

Recently, there has been an increased interest in investigating the role of skeletal muscles in the pathogenesis of NAFLD (65). Sarcopenia is a pathological disorder characterized by generalized loss of skeletal muscle mass and strength. Sarcopenia has been recently proposed as an additional risk factor of NAFLD and a contributor to its development and progression, even after adjusting for body mass index and insulin resistance (66, 67). What was previously only regarded as part of aging (68) is now recognized as a progressive disease frequently associated with cardiometabolic disorders (69). Studies in Asian populations showed that sarcopenia is associated with the presence and severity of NAFLD (66, 70, 71). A prospective study in Western population showed that sarcopenia was associated with the severity of fibrosis and steatosis in NAFLD patients, independently of hepatic and metabolic risk factors (72).

It is now recognized that the muscle is a key metabolic organ and buffers the functions of the liver. While its role as an ammonia buffer is well known with the hepatology community, its role in maintaining body composition is less well appreciated. Muscles primarily utilize free fatty acids in the fasted state and are critical for mobilization of fat stores with diet and exercise. In the metabolically inflexible sarcopenic state, it depends on glucose as its principal substrate for energy and switches to protein breakdown when glucose is not available further contributing to sarcopenia. A host of myokines have been described of which myostatin is the best known (73–75). They both modulate muscle mass and also contribute to a systemic inflammatory state. Systemic levels of cytokines such as Tumor Necrosis Factor-α (TNF-α) and other hepatokines such as Fibroblast growth factor 21 (FGF21) may modulate sarcopenia and muscle function respectively as well. There is thus close cross-talk between the muscles and liver to maintain metabolic homeostasis and its disruption is a key aspect of NAFLD. There are however some caveats in the interpretation of data on muscle structure and function in NAFLD. It is difficult to disentangle the impact of aging on body composition and risk for NAFLD (76), from effects mediated by sarcopenia alone given that aging itself is a risk factor for NAFLD through its association with a decrease in muscle mass, an increase in visceral adiposity, ectopic fat deposition and insulin resistance (66, 67), in addition to more exposure with time to risk factors of NAFLD.

## Metabolic Flexibility and The Role of Adipose Tissue and Muscle

From a pathophysiological perspective, striated muscles play a key role in metabolic homeostasis (77–79). Under physiological conditions, fasting insulin levels are relatively low (80). This releases adipose tissue from insulin-mediated suppression and promotes lipolysis. The free fatty acids released are taken up in striated muscle and used as a principal source of ATP generation. Following a meal, striated muscles clear the circulating glucose load and the muscle shifts from FFA to glucose as a principal source of ATP generation. At the same time, insulin levels rise and suppress lipolysis. This phenomenon of changing fuel source based on availability is also known as metabolic flexibility. Data suggest a relationship between liver and skeletal muscle steatosis in patients with NAFLD. Myokines are active substances derived from skeletal muscle cell. They include myostatin, irisin, myonectin, and various interleukins (IL-6, IL-7, IL-8, and IL-15) and their dysfunction have been implicated in the disrupted adipose–liver–muscle axis in NAFLD (81). Few studies found that skeletal muscle steatosis increased significantly with increasing stage of NASH (82). Fat accumulation in muscles occurs in the context of ectopic fat accumulation and systemic insulin resistance typically associated with NAFLD (82). In the insulin-resistant state associated with obesity, the insulin levels are high and have less variability from fasted to post prandial state. Insulin resistance allows lipolysis to continue in adipose tissue thus releasing excess FFA. While these can be taken up, they are not fully utilized in muscle resulting in ectopic fat storage in the muscle. Also, glucose clearance by muscle is impaired due to insulin resistance. Together, these generate a greater systemic lipid and glucose load which sets the stage for development of excess adiposity and injury to end-organs. In the liver, this is recognized as NAFLD.

## Metabolic Stress to The Liver

Key pathogenic mechanisms driving the progression from hepatic steatosis to NASH include aberrant lipid metabolism, oxidative stress, mitochondrial dysfunction, inflammatory cytokines, immune response and alterations in gut microbiome (83). Lipotoxicity refers to the toxic effects of excessive lipids and lipid derivatives on cells. NAFLD results from delivery of excess FFA and glucose to the liver along with inflammatory cytokines in circulation. Glucose that is not oxidized is converted via de novo lipogenesis in to FFA thus further contributing to the lipid load in the liver. The abundance of FFAs generates cytotoxicity through dysregulation of energy storage homeostasis. Several lipotoxic lipids have been studied, such as FFA, lysophosphatidyl Choline (LPC), ceramides, free cholesterol (FC), and bile acids (BAs). Hepatic steatosis observed in NAFLD occurs secondary to the liver’s attempt to store FFAs in the form of TGs to accommodate excess in FFAs. Adipose tissue derived FFAs are considered drivers of hepatic lipotoxicity. Lipoapoptosis is a principal feature of NASH that results from failure of hepatocytes to dispose of excess FFAs. Intracellular stress leads to hepatocyte apoptosis via activation of intrinsic and extrinsic pathways. The mechanisms involved in lipotoxicity are organelle dysfunctions including endoplasmic reticulum (ER) stress and mitochondrial permeabilization, JNK-induced toxicity and BH3-only protein-induced mitochondrial and lysosomal dysfunction (84–88). Those pathways ultimately lead to activation of caspases mediating apoptosis (44). Further, defects in hepatic mitochondrial fatty acid beta-oxidation have been suggested to contribute to hepatic steatosis and progression to NASH (89). Several studies demonstrated that patients with NAFLD have distinct lipidomic signatures (90–92). NAFLD and NASH are associated with accumulation of highly toxic lipid metabolites (e.g. diacylglycerol, ceramides, sphingomyelin) which trigger inflammation and hepatocyte damage (44, 93). A comprehensive lipidomic analysis on human liver biopsies revealed decreased activity of fatty acid desaturase 1 (FADS1) which is a key player in accumulating toxic lipids during NASH progression (91). The significant complexity of the lipotoxic milieu should be accounted for by targeted therapeutic approaches (94, 95).

## Role of Genetics

Genetics play a key role across the spectrum of NAFLD pathogenesis (96, 97). Variations in genes such as patatin-like phospholipase domain-containing protein 3 *(PNPLA3)*, transmembrane 6 superfamily member 2 *(TM6SF2)*, membrane bound O-acyltransferase domain-containing 7 gene *(MBOAT7)*, glucokinase regulator *(GCKR)*, hydroxysteroid 17-beta dehydrogenase-13 *(HSD17B13)* have been identified as key modifiers of NAFLD development and progression (8). The role of *PNPLA3* gene is discussed in a separate section below. Genetic heterogeneity is involved in various aspects of the disease, namely regulation of energy homeostasis and lipotoxic stress, modulating extracellular matrix production and turnover and controlling inflammation. Studies have shown that genetic variants are implicated in regulating insulin signaling (98), oxidative stress (99), and fibrogenesis (100) thus consequently progression to NASH. 

*TM6SF2* is a gene involved in hepatic very low-density lipoprotein (VLDL) secretion and lipoprotein metabolism. The rs58542926 C>T polymorphism results in loss of this gene’s function or reduction of its hepatic expression, leading to impaired secretion of TG‐rich lipoproteins and increased hepatocyte TG content (96, 101). Studies have shown that mutated variant of *TM6SF2* is associated with development of NAFLD likely through altered hepatic lipid metabolism (102), and higher risk of progression to NASH (103). C-allele is linked with increased cardiometabolic risk (104). A large meta-analysis suggested a correlation between T-allele variant and risk developing T2DM (105). The rs58542926 C>T polymorphism affects nutrient oxidation, glucose homeostasis, and postprandial lipid metabolism and thus contributes to liver injury in NAFLD (106).

*MBOAT7* plays an essential role in the phospholipid remodeling pathway. The rs641738 C>T variant attached to the 3’ untranslated region of MBOAT7 is a commonly associated with a decrease in phosphatidylinositol‐containing arachidonic acid within the hepatocytes which exacerbates liver fibrosis (101, 107). Studies have shown that this MBOAT7 variant is associated with increased risk of NAFLD in Caucasians (108).

*GCKR* controls *de novo* lipogenesis by regulating glucose influx into hepatocytes (109). Loss of function of GCKR protein results in greater hepatic fat accumulation (110). A large NAFLD meta-analysis has identified *PNPLA3* and *GCKR* as factors for increased hepatic TG content (111).

## *Pnpla3* Mediated Progression of Liver Injury And Fibrosis

**Figure 2:**
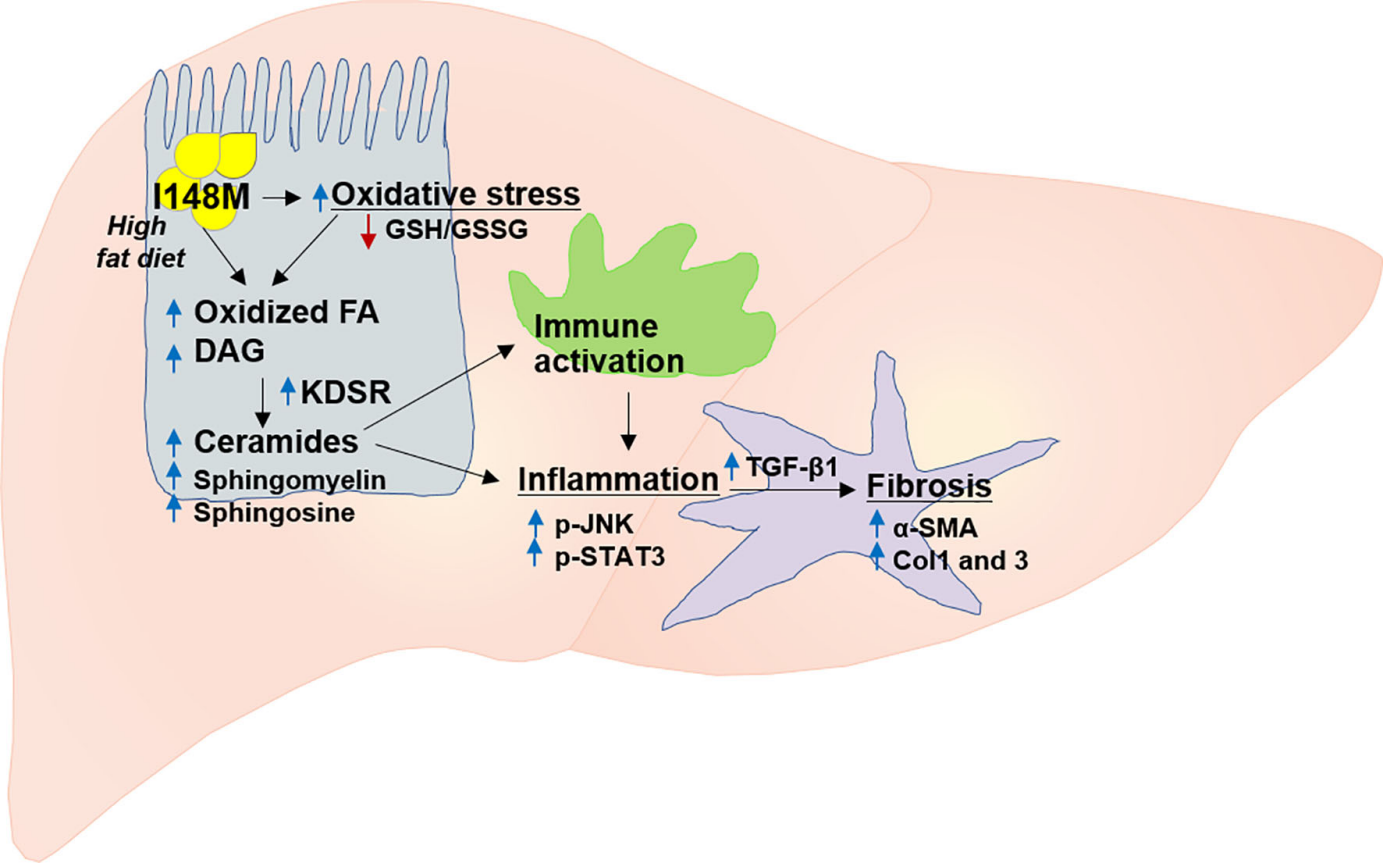
The contribution of mutant *PNPLA3* to the risk of NASH and fibrosis. *PNPLA3* mutation results in increased oxidative stress and fatty acid oxidation which subsequently leads to accumulation of highly toxic lipid metabolites (e.g. diacylglycerol, ceramides, sphingomyelin, sphingosine). These lipotoxic metabolites trigger immune activation and inflammation via key signaling pathways mediated by p-JNK and p-STAT3, and ultimately activation of several fibrogenic pathways such as those for TGF-β1, α-SMA, Col1 and 3. Col1, collagen I; Col3, collagen III; FA, fatty acid; DAG, diacylglycerol; GSH, GSSG, glutathione-disulfide; glutathione; JNK, c-Jun activated kinase; KDSR, 3-ketodihydrosphingosine reductase; SMA, α-smooth muscle actin; STAT3, signal transducer and activator of transcription, TGF-β, transforming growth factor β.

**Figure 3:**
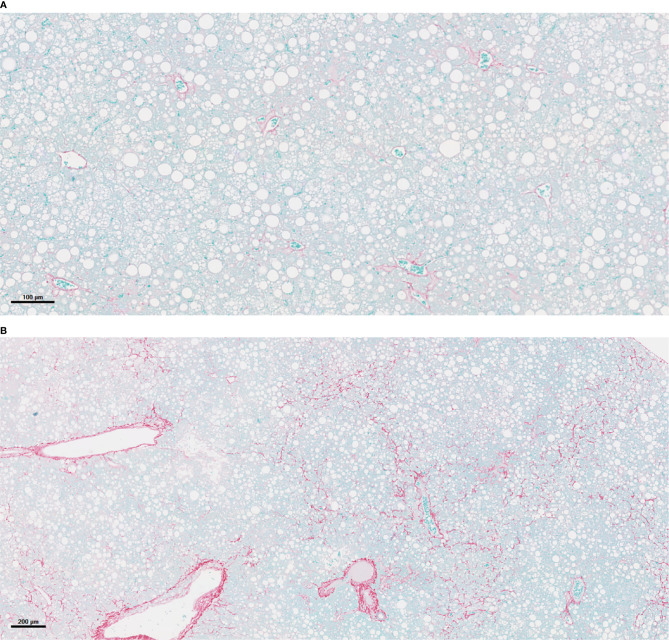
*PNPLA3* ^I148M^-associated acceleration of NASH and fibrosis. **(A)** Liver histology in mouse of empty vector (Luc) after 16 weeks on a Western diet/sugar water (WDSW) diet with NAFLD with minimal fibrosis. **(B)** Hepatocyte of mouse with *PNPLA3* ^I148M^ after 16 weeks on a WDSW diet demonstrating NAFLD with advanced bridging fibrosis. **(A)** 16wks AAV LUC (10x) **(B)** 16wks *PNPLA3* I148M (4x).

*PNPLA3* gene, also called adiponutrin, was the first major gene associated with NASH (112, 113). It is the most replicated modifier of NAFLD pathogenesis in different ethnicities (16). Overall, multiple studies in different populations and diverse ethnicities validated the association between NAFLD and *PNPLA3* (**Figure 2**) *(* 104, 111, 112, 114–116 *).* The *PNPLA3* enzyme is found in hepatocytes and adipocytes and plays a role in lipid remodeling in the liver (108, 109, 112, 117). The wild type *PNPLA3* has function of TG hydrolase and acetyl-CoA-independent transacylase, and loss of its function consequently leads to an accumulation of triglycerides and retinyl esters within hepatocytes (118). Studies have shown that loss of function of *PNPLA3* leads to increased hepatic steatosis and elevated serum alanine aminotransferase (ALT) and aspartate aminotransferase (AST) levels (102, 119). Patients with NAFLD who carry the *PNPLA3* rs738409 C/G mutation, a genetic polymorphism characterized by the substitution of isoleucine to methionine at position 148 (I148M), are more likely to develop steatohepatitis and fibrosis (16), and this nonsynonymous variant is the main genetic risk factor for disease severity and progression (**Figure 3**) (120, 121). Studies consistently showed a strong association between I148M and hepatocellular TG accumulation, increased fibrosis and severity of steatohepatitis (113, 122, 123).

The original studies on mechanisms by which the *PNPLA3* I148M variant led to NASH and fibrosis focused on its function as a triglyceride lipase and the loss of function with this mutation (112). This explained the accumulation of triglyceride but did not explicitly explain how this led to steatohepatitis and fibrosis. Elegant studies by Dr. Hobbs and colleagues further established that with increasing mutant gene expression level, there is an abnormal ubiquitination of the mutant protein with less proteosomal degradation leading to accumulation of the protein on the surface of lipid droplets where its loss of function as a triglyceride lipase further contributed to accumulation of triglyceride in the liver (119, 124). This too does not explain the development of steatohepatitis or fibrosis seen in those with this mutation.

Recently, expression of *PNPLA3* I148M in stellate cells has been related to increased collagen synthesis (125, 126). However, the very low prevalence of significant disease in lean metabolically healthy individuals who carry this mutation makes it unlikely that this alone is enough to explain the impact of this mutation in terms of public health and under conditions of overweight or obesity, those with the mutation appear to have more aggressive fibrosis. A key methodological barrier to studying this question has been the inability to accelerate fibrosis and NASH in a murine model. Whereas silencing *PNPLA3* does not lead to a disease phenotype (127), overexpression of the mutant *PNPLA3*‐148M variant generally has accelerated steatosis and some inflammation but not steatohepatitis with fibrosis.

As demonstrated by several studies, the mechanism seems related to accumulation of *PNPLA3*‐148M on lipid droplets as the 148M variant disrupts ubiquitylation and proteasomal degradation of *PNPLA3* and inhibits other lipases, resulting in impaired mobilization of TG from lipid droplets (119, 124). Animal models showed that *PNPLA3* deletion has no phenotype (128), whereas overexpression or knock-in of the I148M mutation in mice results in increased susceptibility to hepatic fat accumulation (129). A recent study demonstrated that hepatic TG content was associated with significant increase in the *PNPLA3-I148M* (130). A more recent study to evaluate mechanisms underlying *PNPLA3-I148M* induced acceleration of NASH in a murine model where mice received a Western diet with ad lib administration of sugar in drinking water has revealed that under this type of diet, *PNPLA3-I148M* overexpression promotes steatosis and NASH. This study used a model where mice on a high fat high glucose/fructose diet sequentially developed steatosis, steatohepatitis and then progressive fibrosis. This sequential development of lesions was leveraged by testing the hypothesis that introduction of the mutant *PNPLA3* I148M variant but not the wild type *PNPLA3* fed this diet would accelerate the disease and lead to steatohepatitis and fibrosis at a time point where mice with empty vector would only have steatosis. The majority of *PNPLA3*-I148M mice developed severe steatohepatitis with fibrosis (p< 0.0001). In addition, *PNPLA3*-I148M significantly worsened the severity of histological features of NASH including steatosis, hepatocellular ballooning and lobular inflammation (p< 0.001). The principal observation in this study is that the hallmark feature linked to *PNPLA3-*I148M-induced NASH acceleration is ‘metabolic reprogramming’ of the liver with increased TGs and diglycerides, n3 polyunsaturated fatty acids depletion and increased ceramides. *PNPLA3*-I148M also had a significant impact on several cellular processes including proteosomal, phagosomal, and lysosomal function, amino-acyl T RNA synthesis, circadian rhythm and ER function. *PNPLA3*-I148M expression promoted cell death and increased oxidative stress and ER stress. Multiple inflammatory pathways were activated with *PNPLA3*-I148M, such as JAK-STAT system. Specifically, the STAT3 system was activated significantly in the presence of the I148M variant. Integrated pathway analysis revealed a strong signature of ceramide related inflammatory activation including h-ras and downstream PLA2 activation, suggesting a key role for sphingolipids in the inflammatory response to *PNPLA3*-I148M. Furthermore, *PNPLA3*-I148M was associated with activation of several fibrogenic pathways such as those for Procollagen I, III and α-smooth muscle actin mRNA, as well as increased transforming growth factor beta (TGF-β1) protein levels (131). Importantly, this accelerated phenotype of disease could be rescued by silencing the mutant *PNPLA3* I148M variant.

The findings reported in animal models were also supported by human genetic studies which showed that *PNPLA3* rs2294918 E434K variant decreased *PNPLA3* expression, reducing the effect of the I148M variant on the predisposition to steatosis and liver damage (127). This suggests that the *PNPLA3* I148M variant expression is required for *PNPLA3*‐associated hepatic steatosis by the aforementioned mechanism. These data suggest that the mutant *PNPLA3*-I148M protein can be a new therapeutic target for NAFLD and NASH (132).

## Summary and Future Directions

A growing body of literature indicates that the development of the NAFLD phenotype (fatty liver or steatohepatitis) and its progression to cirrhosis represents a complex set of gene-environment interactions. While environmental factors such as changes in diet play a central role, the role of other factors such as environmental pollutants are also becoming apparent and worthy of further investigation.

The role of genetics in modulating the metabolic drivers of NASH will be a major area of research over the next decade. Following the landmark studies identifying a linkage between the I^148^M variant of *PNPLA3* and progressive NASH, an increasing number of genetic variants have also been linked to the disease. However, for several of these the specific mechanisms by which they affect NASH have not been clarified. Particularly the HSD17B13 splice variant which has a protective role is an important new discovery and future studies will be needed to define how it contributes to the disease and even potentially negates some of the deleterious effects of the *PNPLA3* mutation.

It is further clear that individual mutations can not only have an impact based on their biological effect but multiple mutations in different genes in various metabolic pathways can interact to modulate the metabolic response to the lipotoxic load being delivered to the liver. Development of a rigorously validated polygenic risk score remains an important unmet need in the field.

A key focus of this manuscript has been the growing body of literature on the role of the extrahepatic milieu as a cause of the liver disease. Much more work needs to be done on the relationship between changes in diet and the changes in intestinal epithelium and how these alter the meal stimulated gut hormonal response. This is likely to be a key modulator of the state of metabolism and the development of both the metabolic syndrome and fatty liver disease. Early data from the metabolic benefits of duodenal mucosal resurfacing provide a strong rationale to continue to pursue this line of research. These are also likely to yield potential therapeutic approaches beyond duodenal mucosal surfacing.

The role of the intestinal microbiome continues to be elucidated as well. There is already a plethora of literature demonstrating changes in specific taxa that are linked to the NASH and even advanced fibrosis. While some data on metagenomics are available, they are not tightly concordant with the metabolomic signatures in stool and direct interrogation of the microbial transcriptome will be needed to better understand the role of altered microbial taxa in NASH. Further functional analyses of changes in the microbiome will provide mechanistic insights into the nature of microbial contribution to the disease and its progression. It will also set the stage for phase-based therapeutics for NASH.

Additional intestinal factors such as secondary bile acids are likely to also be important. Many bacteria are susceptible to the antibiotic functions of secondary bile acids and decrease when secondary bile acids increase whereas bacteria that require such bile acids for growth increase. These may provide a potential explanation for the changes in microbial composition. Further systemic uptake of secondary bile acids may have direct effects on metabolism via FXR, TGR5 and affect processes such as senescence to cause hepatocellular cancer.

Finally, the importance of striated muscle and adipose tissue in driving NASH is also becoming apparent and direct modification of muscle function by specifically engineered diets designed to break metabolic inflexibility to improve the systemic metabolic state are likely to improve not only NAFLD but the systemic state of dysmetabolism.

In summary, while much progress has been made in understanding the metabolic drivers of NASH, much additional work remains to be done. These are likely to have a major impact on the understanding of the role of the systemic changes in metabolic syndrome and how these lead to NASH. Gene-environmental interactions and studies to define subpopulations based on clustering of specific genetic and environmental factors may provide insights on the heterogeneity of NAFLD and new approaches to treat individual patients.

## Author Contributions

Both authors contributed to the writing of the manuscript. All authors contributed to the article and approved the submitted version.

## Conflict of Interest

AJS is President of Sanyal Biotechnology and has stock options in Genfit, Akarna, Tiziana, Indalo, Durect, Exhalenz and Hemoshear. He has served as a consultant to Astra Zeneca, Conatus, Coherus, Bristol Myers Squibb, Blade, Tobira, Takeda, Siemens, Merck, Genentech, Tern, Gilead, Lilly, Poxel, Artham, Boehringer Ingelhiem, Novo Nordisk, NGM Bio, Birdrock, Novartis, Pfizer, and Genfit. He has been an unpaid consultant to Intercept, Echosens, Perspectum, Immuron, Galectin, Fractyl, Affimune, Chemomab, Nordic Bioscience. His institution has received grant support from Gilead, Salix, Tobira, Intercept, Bristol Myers, Shire, Merck, Astra Zeneca, Malinckrodt, Cumberland and Novartis. He receives royalties from Elsevier and UptoDate.

The remaining author declares that the research was conducted in the absence of any commercial or financial relationships that could be construed as a potential conflict of interest.
